# Peripheral Sensory Stimulation for Long-Term Improvement in Mild Cognitive Decline: A Prospective Interventional Study

**DOI:** 10.3390/brainsci16030265

**Published:** 2026-02-27

**Authors:** Tom Zhang, Fei Sun, Andre Stang, George Ayoub

**Affiliations:** 1 Department of Chinese Medicine, Wuhe Chengnan Hospital, Bengbu 233300, China; 2 School of Social Work, Michigan State University, East Lansing, MI 48823, USA; 3 Department of Development and Science, Sili-Tec GmbH, 26135 Oldenburg, Germany; 4 Department of Psychology, Santa Barbara City College, Santa Barbara, CA 93109, USA

**Keywords:** cognitive decline, PISTA, tactile stimulation, dementia, pain

## Abstract

Background: Despite recent breakthroughs in pharmacological treatment for Alzheimer’s disease, high costs and the complex procedure to monitor safety have limited access for many patients. Less invasive and more accessible non-pharmacological therapies that support neuroplasticity and slow cognitive decline are needed. Processing Inner Strength Toward Actualization (PISTA) stimulation applies structured tactile input to promote cortical–subcortical activation. This study evaluated the long-term effects of PISTA on cognition and pain in older adults with mild cognitive impairment or early dementia. Methods: This single-arm, prospective trial enrolled 100 outpatients aged 47–70 years at outset (50 women, 50 men) with no control group. Participants received clinician-supervised PISTA stimulation three times weekly for 48 months. Each 30 min session delivered rhythmic tactile input calibrated to individual sensory thresholds. Cognitive performance was assessed monthly using the Mini-Mental State Examination (MMSE). Perceived pain was measured monthly with the Numeric Pain Rating Scale. Outcomes were analyzed using ANCOVA, adjusting for age, sex, and baseline cognitive status. Results: Cognitive scores improved significantly across all age strata, with a mean annual MMSE increase of 0.75 points (95% CI: 0.26–1.21; *p* < 0.0025). Pain intensity decreased in parallel (mean reduction: 0.56 points; 95% CI: 0.34–0.78; *p* < 0.001). Improvements in cognition and pain were moderately correlated (r = 0.38). The greatest combined benefits occurred in participants aged 55–62 years. No serious adverse events were observed during the 48-month trial. Conclusions: PISTA stimulation produced sustained improvement in cognition and reduced perceived pain, supporting its promising role as a safe, non-invasive adjunct for neurodegenerative cognitive decline. These findings suggest peripheral sensory activation as a promising driver of functional neuroplasticity and warrant verification in randomized, controlled trials.

## 1. Introduction

### 1.1. Background

Non-pharmacological interventions have garnered increasing interest as safe, effective treatment options to slow or reverse cognitive decline and support functioning among older adults. With the limitations (such as high costs and modest benefits) and adverse effects (such as an increased risk of intercranial bleeding) of pharmacological therapies for Alzheimer’s disease and related dementias, alternative approaches have become a critical focus in research and in clinical care [1,2,3]. Recent meta-analyses and systematic reviews have demonstrated that modalities such as physical exercise, cognitive stimulation therapy, music therapy, and social engagement exert meaningful benefits on the cognition, functional status, and psychological well-being of individuals at risk of or experiencing mild cognitive impairment or dementia [3,4,5,6].

A study of 2000 older adults with a sedentary lifestyle found that a structured intervention was more effective than self-guided interventions in reducing cognitive decline over the course of two years [7]. This report included cognitive games and showed that the structure was essential to determining the effect. Another study analyzed data from a UK aging cohort and found a protective function in those who reported playing musical instruments throughout their lives [8]. This provides further evidence that structured activities are more beneficial, as musicians report practicing for extended periods each day. Additionally, the mechanical movements of playing provide improved dexterity, and the music provides aural stimulation. Related evidence has been previously reported in a meta-analysis of trials with dementia patients, as music therapy has been found to be effective in improving cognitive functions, with a greater impact when the patients were involved in making the music [9]. Additionally, another review of the benefit of music on cognition found a specific tie to rhythmic stimulation, explaining that the benefit appears to be due to the patients’ attempt to synchronize their actions, which may be bridging music therapy and kinesthetic therapy [10]. Supporting this kinesthetic/music enhancement is a recent review of arts-based therapy, which reports that dance is another activity that benefits cognition [11]. In a striking report on 45 people with severe dementia, individualized music listening was found to be beneficial, and engagement in synchronous activities while listening to music was recommended to maximize these benefits [12].

Thus, there is a history of using coordinated sound and movement to improve cognition in those with mild and even advanced cognitive decline. There is also evidence that cardiovascular therapies are beneficial. There is an association with diminished cardiovascular health and increased dementia, especially as seen in patients with diabetes [13]. But more centrally, multiple studies have pointed to the cognitive benefit of physical exercise, as seen in a recent review of such work [14].

Emerging research has highlighted the potential of novel sensory-based approaches, including sound and vibrational therapy, to enhance neuroplasticity and mitigate age-related decline [7]. The PISTA (Processing Inner Strength Toward Actualization) system is a pioneering example of this modality, using auditory-based entrainment along with personalized sensory interventions to improve cognitive function and reduce psychosocial distress in older adults with dementia [15,16]. Early pilot and case-series data have reported significant reductions in behavioral and psychological distress and improvement in well-being among both individuals with cognitive decline and their caregivers.

This case series explores a four-year review of the application of the PISTA system in a clinical context, presenting real-world outcomes and insights from individuals treated for psychological distress and cognitive decline using this innovative, non-pharmacological approach [16]. By situating PISTA among established and emerging treatment modalities, this report aims to advance our understanding of sensory-based interventions for dementia and contribute to the evolving evidence base on non-drug strategies for cognitive health in aging populations.

### 1.2. Research in Context

#### 1.2.1. Evidence Found Before This Study

We searched PubMed, Scopus, and ClinicalTrials.gov for studies (“cognitive decline”, “sensory stimulation”, “tactile therapy”, and “somatosensory activation”) published between 2000 and June 2025. Evidence indicates sensory and tactile interventions can transiently improve attention and pain in neurodegenerative conditions, but no longitudinal studies employing structured PISTA stimulation and monthly cognitive and pain monitoring were identified.

#### 1.2.2. Added Value of This Study

This is the first prospective, structured PISTA stimulation study longer than 12 months targeting patients with mild cognitive impairment or in the early stages of dementia, with monthly blinded assessments of cognition (MMSE) and perceived pain (Numeric Pain Rating Scale). The standardized protocol, adherence monitoring, and clinical supervision of this study support its replicability and safety.

#### 1.2.3. Implications of All the Available Evidence

This study supports peripheral sensory activation via PISTA stimulation as a novel, non-invasive method to modulate neuroplasticity within somatosensory and cognitive networks in neurodegenerative disease. Sustained cognitive improvements and reduced pain suggest sensory input influences the CNS circuits involved in cognition and nociception. Integration of PISTA into neurorehabilitation could complement pharmacologic and behavioral therapies. Future randomized trials incorporating neuroimaging and biomarkers are essential to elucidate mechanisms and optimize clinical use.

## 2. Materials and Methods

### 2.1. Study Design and Oversight

This study was a prospective, single-arm interventional trial conducted according to the TREND statement for reporting non-randomized interventions for behavioral and public health interventions. The objective was to evaluate the effects of Processing Inner Strength Toward Actualization (PISTA) stimulation on cognitive decline and perceived pain in adults with mild cognitive impairment or in the early stages of dementia. The protocol was approved by the Institutional Review Board of Wu He Chengnan Hospital, Anhui, China (approval no. EC-WHC-2021-001). Written informed consent was obtained from all participants or their legal representatives.

### 2.2. Participants

Participants aged 47–70 years with a diagnosis of mild cognitive impairment or early-stage dementia per DSM-5 and NIA-AA criteria were recruited at Wu He Chengnan Hospital, Anhui, China in 2021 and were followed for 48 months. Exclusion criteria included unstable systemic illness, major psychiatric disorders, significant peripheral neuropathy, recent cerebrovascular events, active malignancy, and concurrent participation in other interventional studies. Information about baseline demographics, comorbidities, medications, and cognitive status were collected. The first 50 women and first 50 men seen by the clinics in 2021 were the ones included in this study, with age categories of 47–54 (n = 17 female, 17 male), 55–62 (n = 17 female, 14 male), and 63–70 (n = 16 female, 19 male). We note our sampling of the first 50 qualified candidates may introduce some selection bias in this pilot study.

### 2.3. Intervention

The PISTA intervention consisted of structured audio and tactile stimulation sessions targeting peripheral afferent nerves to engage somatosensory cortical networks. The PISTA Life Power device [16] was employed for each patient and placed around the patient’s neck. The PISTA Life Power device uses slow rhythmic modulations (0.52–1.30 Hz) that fall primarily in the delta range (0.5–4 Hz) of human brain activity, which is commonly associated with deep relaxation, recovery, and restorative physiological states. This low-frequency stimulation may promote neural entrainment, whereby external rhythmic input biases ongoing cortical oscillations toward slower patterns linked to reduced arousal and pain perception.

Participants underwent three 30 min sessions per week over 48 months, delivered by clinicians trained in use of PISTA. Stimulation intensities were individualized based on baseline sensory threshold assessments and adjusted when discomfort occurred. Device calibration and maintenance complied with manufacturer and biomedical engineering standards.

Patients were followed up between visits by phone to help support steady adherence to the protocol.

### 2.4. Outcome Measures

Primary outcomes were monthly cognitive scores assessed by trained clinicians blinded to previous results, using the Mini-Mental State Examination (MMSE). Patients were blinded, and we ensured they were not informed of their assessment scores during their visits. The secondary outcome was perceived pain intensity rated monthly on a Numeric Pain Rating Scale by clinic staff during visits. The MMSE and pain measures were primarily used as screening measures rather than diagnostic tools, and the study was not designed or powered to formally evaluate minimal detectable change or minimal clinically important difference. Exploratory outcomes included quarterly self-reported mood, energy, and daily functioning assessed by standardized questionnaires.

### 2.5. Data Collection and Management

Data were recorded in secure, password-protected electronic case report forms compliant with Good Clinical Practice. Quality controls included double data entry, automated missing-value checks, and periodic audits against source documentation. Data analysts were blinded to participant identifiers until primary analyses were completed.

### 2.6. Analysis Strategies

Analysis followed a modified intention-to-treat approach and included all participants receiving at least one PISTA session. Longitudinal changes in cognition and pain outcomes over the 48-month follow-up were recorded monthly and grouped by age and gender, resulting in three groupings for men and for women, to depict any variation due to age category or gender. Group averages were used to assess impact by cohort, and individual records were evaluated to identify adherence of each cohort member to determine the impact of PISTA by cohort.

### 2.7. Trial Registration and Ethical Compliance

The study conformed to the Declaration of Helsinki and Good Clinical Practice guidelines. The protocol and informed consent procedures received Institutional Review Board approval. Informed consent was obtained from all participants or legal representatives, and data were anonymized for confidentiality. The study’s sponsors had no influence on how the study was conducted or the analysis.

## 3. Results

**Figure 1:**
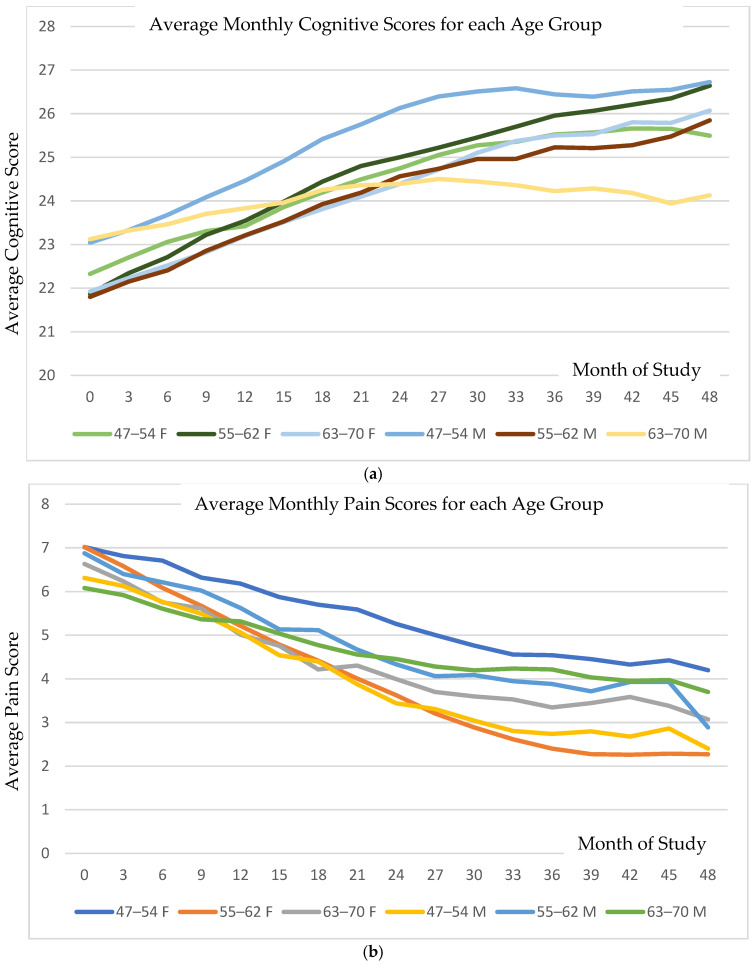
(**a**) Illustration of the monthly average cognitive scores (with a range of 1–30) and (**b**) perceived pain scores (on a 0–10 scale) across study groups over the treatment duration in months. Although the average scores increased incrementally, detailed examination reveals that the rate of improvement in both cognition and pain was consistent among responders, defined as individuals with ≥1-point increase in cognitive score or ≥1-point decrease in pain score.

Figure 1a shows the average monthly cognitive scores for each age group (on a scale of 1–30), and Figure 1b shows the average perceived pain data for the same groups (on a scale of 1–10). Each category started with a baseline cognitive score near 22–23 on average, indicative of cognitive decline. On average, each group showed increasing scores for the first two years, with most averages reaching a plateau that was 3.5–4 points higher, at 25.5–27, with the exception of the older males, whose scores increased by one point. In both graphs, the horizontal axis is the time (months) since the onset of the treatment. Cognitive scores improved significantly across all age strata, with a mean annual MMSE increase of 0.75 points (95% CI: 0.26–1.21; *p* < 0.0025). Pain intensity decreased in parallel (mean reduction of 0.56 points; 95% CI: 0.34–0.78; *p* < 0.001). Improvements in cognition and pain were moderately correlated (r = 0.38).

The averaged data obscure that the rate of improvement in cognitive or pain score was a constant for all who responded to the PISTA intervention. Responders were defined as each individual having a positive change of one point or more in cognition or negative change of one point or more in pain.

**Figure 2:**
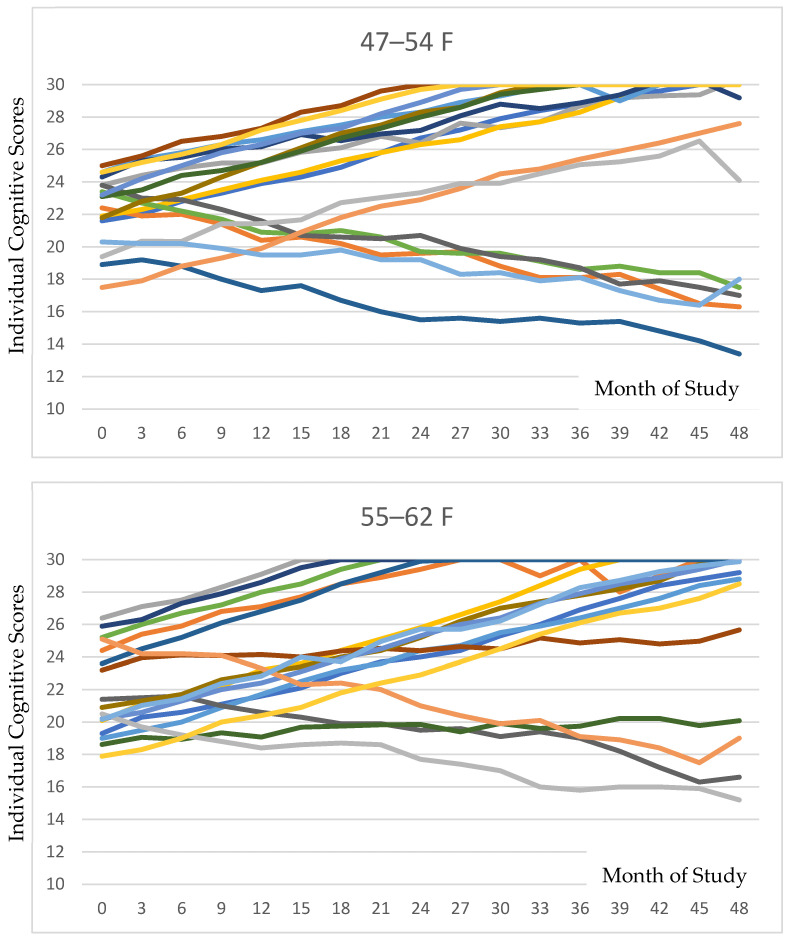

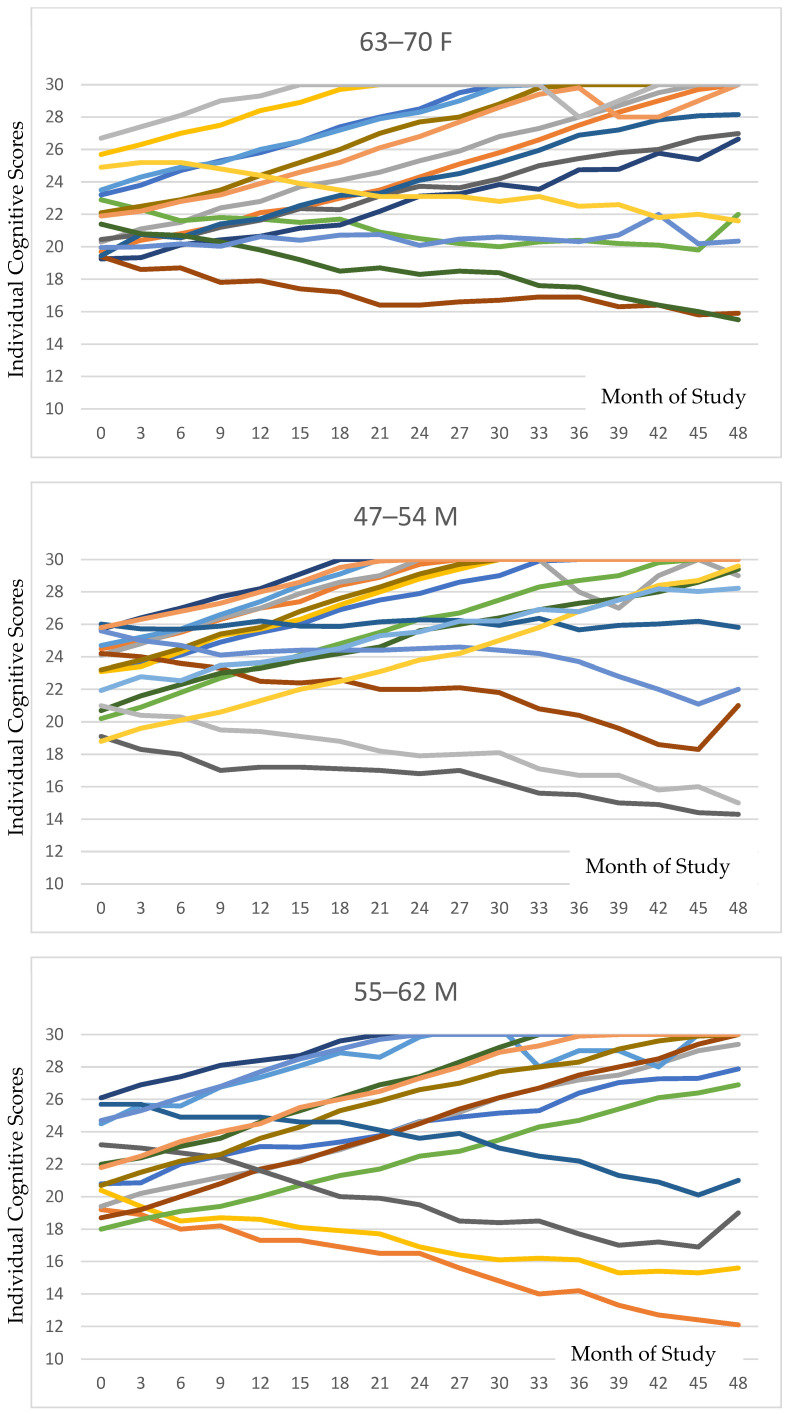

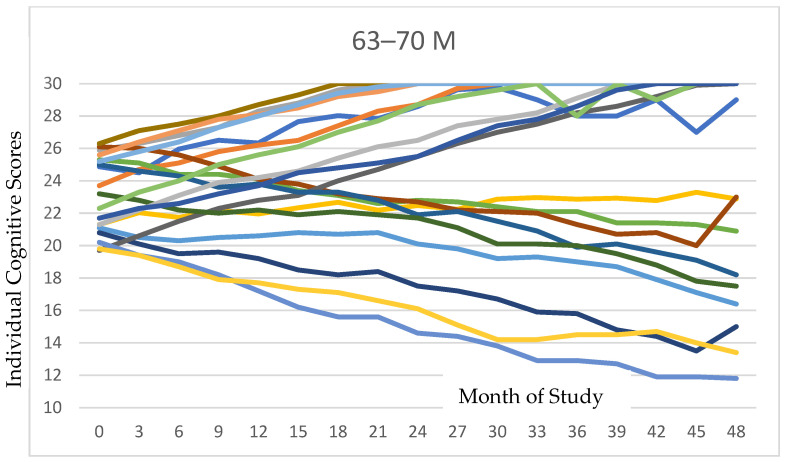
Individual cognitive trajectories stratified by age and sex are depicted for each of the six groupings. As is evident in the tracings, the majority of participants in each subgroup exhibited positive responses to PISTA stimulation.

Figure 2 presents all individual data for cognition for each age and gender category. The variations among individuals can be delineated in these data.

Evaluating the data, we observed that the majority of individuals within each group had a positive response to the PISTA intervention. The percentages with positive response in each group are depicted in Table 1, with the fourth column in the table indicating the percentage of participants who had a positive response in both categories. The final column depicts the average annual change in cognitive score in the first 15 months. The period of fifteen months was chosen due to some individuals reaching a maximum cognitive score at 15 months. The data shown are annualized. Cognitive improvement for responders in each group was found to be near 2.5 for those under 63 years and 2.25 for those over.

**Figure 3:**
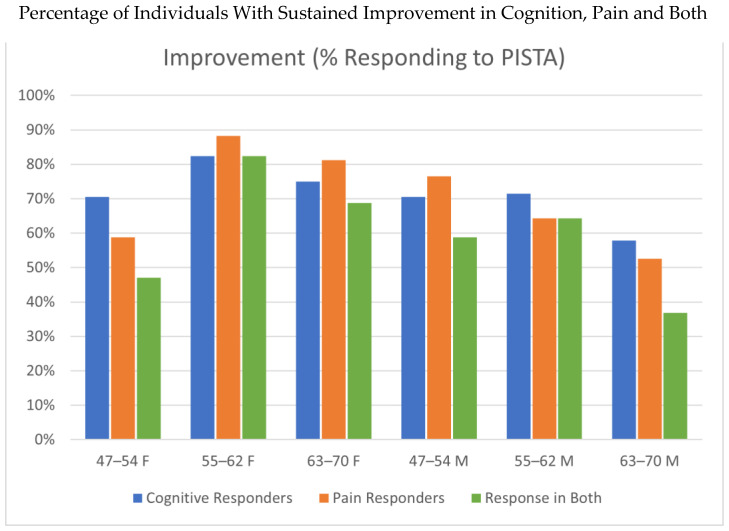
Composite responder data, showing proportions achieving at least a 1-point improvement in cognition (blue bars), pain (orange bars), and in both domains concurrently (green bars). While consistent response rates occurred in the middle-aged groups, the youngest female cohort (47–54 years) and the oldest male cohort (63–70 years) demonstrated less of an overlap between cognitive and pain improvements.

Figure 3 is a composite of the data of responders, showing the percentages of those having a minimum of a one-point improvement in cognitive score (blue bars), pain score (red bars), and those individuals who had an improvement in both scores (green bars). While the middle-aged category showed consistency in responders, the youngest age group of women and oldest age group of men revealed the lowest correspondence of cognition and pain responders.

## 4. Discussion

This clinical trial investigated the long-term effects of PISTA stimulation on cognition and pain perception in individuals with mild cognitive impairment and early dementia. Over the course of 48 months, consistent cognitive improvement and pain reduction were observed, particularly during the first 15 months of treatment. These findings support the hypothesis that peripheral sensory activation can sustain neuroplastic changes in cortical and subcortical circuits associated with cognitive and affective processing.

### 4.1. Interpretation of the Findings

The gradual increase in cognitive performance and decline in perceived pain across participants are consistent with neuronal plasticity induced by continuous sensory engagement. Peripheral tactile activation is known to influence central network activity via thalamocortical pathways, enhancing cortical excitability and modulation of attention networks. Previous studies of neuromodulatory sensory stimulation, including auditory and vibrotactile therapies, have shown transient improvements in visuospatial and working memory outcomes [17]. We suggest that the persistence of effects in this trial suggests that sustained exposure to multisensory tactile input may help strengthen synaptic integrity and/or stabilize cognitive networks involved in executive and mnemonic functioning. Potential pathways for such change might be the activation of somatosensory afferents, modulation of arousal and attention networks, and/or potential downstream effects on neural plasticity. These are mechanisms we propose to examine in future tests.

We note that the two groups with the lowest coherence between cognitive and pain improvement were the younger female category (with an age of 47–54 at onset) and older male category (with an age of 63–70 at onset). All other groups had well over 60–80% of their members responding in terms of both cognition and pain. It would be valuable to identify if there are nutritional deficits in either of these two outlier groups that may be limiting the improvement in their cognitive scores [18]. We have also taken into consideration that the cognitive decline in a larger fraction of either of these categories might have meant that the members may have neglected to follow the PISTA protocol at home or inaccurately recalled following such procedure. In reading the individual data (Figure 2), it seems clear that a larger number of members in each of these groups showed gradual cognitive decline, as compared to the individuals in the other groups, while responders in these two groups showed similar rates of improvement to the other groupings. We thus conjectured this might reflect a dietary deficiency or a compliance limitation.

### 4.2. Relation to Previous Research

These results extend an expanding body of evidence demonstrating the beneficial effects of non-pharmacological interventions on cognitive decline [1,2,3,4,5]. While prior systematic reviews have established that cognitive and physical training can modestly enhance or preserve function in dementia, few interventional studies have targeted the somatosensory system directly. Of importance, our study spanned four years. As can be observed in the individual data reports (Figure 2), the improvement in cognitive scores typically occurred in the first year or two. We believe our study expands on other works, and shows that the continuation of the PISTA protocol revealed a sustained cognitive benefit, with very few participants showing any decline even after three years into the study. Thus, a key point, that of sustained benefit, is answered in our study here. The use of PISTA therapy resulted not only in an improvement in cognitive and pain scores, but also this improvement was maintained for the following years.

Sensory activation methods such as those in the PISTA protocol differ from previously trialed behavioral or exercise programs by closely integrating peripheral afferent feedback within a repetitive neuromodulation schedule. This integration may explain the concurrent improvements observed in both cognitive and pain domains, as nociceptive and cognitive circuits are reciprocally modulated through shared prefrontal–limbic pathways.

### 4.3. Clinical and Translational Implications

The safety profile and feasibility of regular clinic-based PISTA sessions indicate strong potential for translation to community and rehabilitation contexts. Non-pharmacological modalities can complement pharmacotherapy in early dementia by modulating cortical function without the side-effect burden of conventional drugs. Implemented as part of a multidomain care model, tactile activation could help extend functional independence and reduce caregiver stress, a conclusion consistent with earlier multimodal intervention trials showing synergistic effects on cognition, mood, and quality of life [5,6].

### 4.4. Strengths and Limitations

This longitudinal study’s strengths include its frequent, blinded cognitive and pain assessments, structured intervention regimen, and long duration of follow-up. Inclusion of both men and women across defined age strata enables the exploration of sex- and age-related differences in neuroplastic responsiveness, a growing priority in dementia research. However, the absence of a randomized comparison group limits causal inference. The open-label design may introduce expectancy bias, and lifestyle confounders cannot be fully excluded. Additionally, because minimal clinically important difference thresholds for this population and measurement context were not applied a priori, the clinical significance of this change cannot be definitively determined.

The use of a single-arm, open-label design without a control or comparison group limits causal inference. Future randomized controlled trials that address this and ones with alternative sensory control conditions are needed. Neuroimaging and neurophysiological measures would also be valuable for confirming cortical changes and identifying neural correlates of response.

In addition, the participant sampling method used for the first qualifying patients may have introduced a bias in this pilot, which needs to be addressed with population-based or systematic sampling methods in future trials to strengthen its external validity.

### 4.5. Future Directions

The observed parallel improvement in cognition and pain underscores the interconnectedness of sensory and cognitive processing. Mechanistic studies using functional MRI and quantitative EEG could clarify how PISTA influences sensory–cognitive circuits, especially in dopaminergic and cholinergic systems implicated in attention and memory. Comparative studies with non-invasive neuromodulation techniques such as transcranial magnetic or vagus nerve stimulation could further define whether tactile interventions operate through distinct or convergent pathways. Longitudinal translation studies in other neurodegenerative and pain syndromes may extend these findings to broader patient populations.

## 5. Conclusions

Our results indicate that structured tactile activation through PISTA stimulation is associated with sustained improvement in cognitive and pain outcomes, supporting its potential as a safe, replicable, and low-cost adjunct to conventional dementia care. This study shows that an easy-to-administer, non-invasive sensory stimulation intervention can help slow cognitive decline and reduce pain among individuals with mild cognitive impairment and early-stage dementia; a population for whom effective, accessible quality-of-life interventions remain limited. By engaging putative sensory–cortical pathways, PISTA may provide a biologically plausible mechanism for reversing functional decline and warrants confirmation in controlled multicenter trials.

## Figures and Tables

**Table 1:** Proportion of responders are depicted by category, including those who improved in terms of both cognition and pain. The final column of the table presents the annualized mean change in cognitive scores over the initial year, a time frame selected due to some participants reaching maximum cognitive assessment scores within 15 months. Among responders, mean annualized cognitive improvement was approximately 2.5 points for participants under 63 years and 2.25 points for those aged 63 and older.

Age/Gender	Percent Pos. Changed	Annualized Rate
47–54 F	71%	2.37
55–62 F	82%	2.38
63–70 F	75%	2.14
47–54 M	71%	2.57
55–62 M	71%	2.55
63–70 M	58%	2.34

## Data Availability

The data presented in this study are available on request from the corresponding author due to privacy restrictions.

## Abbreviations

The following abbreviations are used in this manuscript:

PISTA	Processing Inner Strength Toward Actualization
MMSE	Mini-Mental State Examination
CNS	Central Nervous System
DSM-5	Diagnostic and Statistical Manual of Mental Disorders, Fifth Edition
NIA-AA	National Institute on Aging and Alzheimer’s Association
